# Influence of lead on the activity of soil microorganisms in two Beskidy landscape parks

**DOI:** 10.1007/s10661-021-09503-2

**Published:** 2021-11-24

**Authors:** Jacek Borgulat, Włodzimierz Łukasik, Anna Borgulat, Aleksandra Nadgórska-Socha, Marta Kandziora-Ciupa

**Affiliations:** 1 Institute for Ecology of Industrial Areas, Kossutha 6, 40-844 Katowice, Poland; 2 Department of Water Protection, Central Mining Institute, pl. Gwarków 1, 40-166 Katowice, Poland; 3 Department of Ecology, University of Silesia, Bankowa 9, 40-007, Katowice, Poland

**Keywords:** Heavy metals, CLPP, Soil enzymes, Soil respiration

## Abstract

The aim of the study was to assess the potential impact of lead on soil metabolism in two landscape parks localized in the Beskid Śląski and Beskid Żywiecki mountains which were affected, among others, by air pollution from the Upper Silesian Industrial Region, the largest industrial zone in Poland. The study was carried out in six locations with different lead levels in the soil environment. Each plot was equipped with four pairs of vacuum ceramic lysimeters to assess the mobility of Pb in the soil. The metabolic activity was assessed by measuring: soil enzyme activity, soil respiration and by studying community-level physiological profiling (CLPP) using Biolog EcoPlates technique. The soil to the examination was collected near the stands with the lysimeters from two soil horizons (A and B layer). The analyses carried out showed that the factors that had the greatest influence on lead mobility were the organic carbon content and the soil pH. The elevated lead level in the topsoil (layer A) could affect the functional biodiversity of soil microorganisms, but low soil pH was a more likely limiting factor. In the subsoil (layer B), lower lead content was found and its probable effect on soil microbial activity was small. In summary, it can be concluded that the assessment of the influence of heavy metals on soil metabolism is not easy, and the Biolog system has proven to be a sensitive tool for assessing the potential impact of heavy metals on the soil environment.

## Introduction

Over the years, the Beskidy region was subjected to strong anthropopressure from the surrounding industrial areas (Kłos et al., 2018; Uziębło et al., 2012). It was found that the pollution came mainly from the area of Ostrava and Upper Silesia. This thesis is confirmed by the decrease in the amount of pollutants reaching forest ecosystems, including heavy metals and acidic compounds, noticeable since 2000s, when many industrial plants began to close down on the mentioned areas (Staszewski & Kubiesa, 2008; Staszewski et al., 2008). An ecological disaster has been taking place in the Beskidy for many years. It is believed that air pollution and other factors such as progressive soil acidification, pathogens, inadequate planting material, and drought could have had an impact on the dieback of spruce — most common tree in the Beskidy mountain forests. However, there is a lack of information about the possible impact of deposited pollutants like heavy metals including lead on the other parts of environment like the soil microflora.

It should be remembered that the soil microorganisms are the most important factor conditioning the availability of nutrients for plants, enabling them to grow and develop. Knowledge about the influence of heavy metals on soil microorganisms is still insufficient. It is known that these elements, if background level is exceeded, can disturb the homeostasis in the soil environment, limiting biodiversity and the number of microorganisms. The harmful effect of lead and other heavy metals largely depends on their mobility in the environment and bioavailability, which is influenced by many factors such as environmental acidity, organic matter content, and soil granulometric composition (Kaczynska et al., 2014).

The use of biological and biochemical parameters to assess the ecological status of environmental samples provides accurate information (Gryta et al., 2014). One of the commonly used methods in studying the functional diversity of microorganisms is the Biolog EcoPlate technique. This method was used to assess stressing impact on soil environment like pH and salinity (Pankhurst et al., 2001) and also the toxicological impacts of pollutants on it such as hydrocarbons (Nagy et al., 2013) and heavy metals (Boshoff et al., 2014; Feigl et al., 2017). Other monitoring research (Huang et al., 2017) showed that the effect of soil additives on community-level physiological profiles (CLPP) were correlated with the sequencing results like 16S rRNA and ITS rRNA. The research of soil enzymatic activity was the source of information about the condition of soil environment (Telesiński et al., 2019) and also the changes occurring in it (Trasar-Cepeda et al., 2008). 

The aim of the study was to assess the impact of lead on the activity of soil microorganisms in the Beskid Śląski and Beskid Żywiecki mountains by using various methods.

## Materials and methods

### Site description, sampling, transport, and storage of samples

**Fig. 1:**
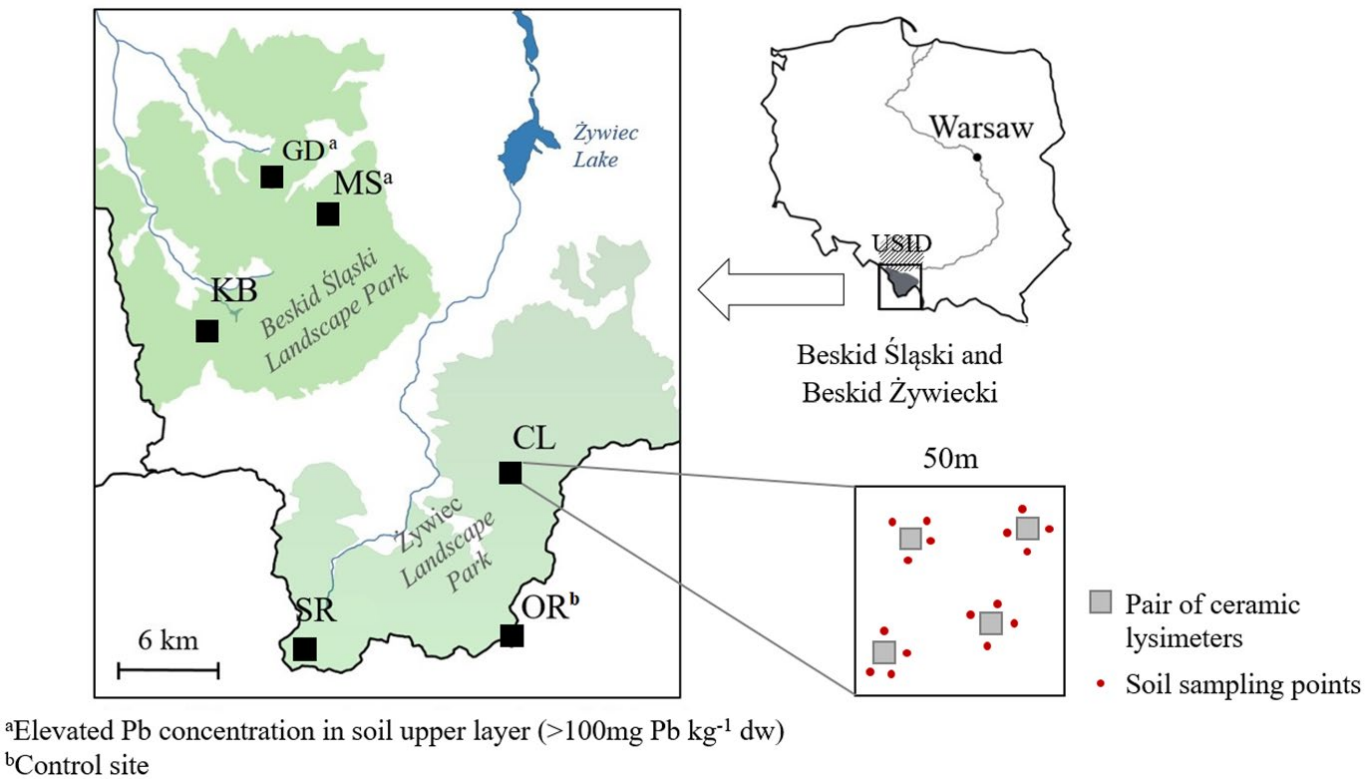
Localization of sampling sites. CL, Czarny Las; GD, Godziszka; KB, Kubalonka; MS, Małe Skrzyczne; OR, “Oszast” nature reserve; SR, “Śrubita” nature reserve. USID, Upper Silesian Industrial District

Samples were collected from six localizations in the Beskid Śląski and Beskid Żywiecki mountains. Three sites were localized in Beskid Śląski Landscape Park: GD, Godziszka; KB, Kubalonka; MS, Małe Skrzyczne, and another three in Żywiecki Landscape Park: CL, Czarny Las; OR, “Oszast” nature reserve; SR, “Śrubita” nature reserve (Fig. 1). The OR site located in the southern part of the Żywiec Beskidy was considered a control plot due to its low lead load in the soil. All sites were located in mixed spruce–fir–beech mountain forests at an altitude of between 600 and 800 m above sea level. Each site was previously equipped with 4 pairs of vacuum ceramic cup lysimeters for sampling of soil solution. The cups were placed in the A and B soil layers. Soil solutions were collected at least twice a month in the period from April to November 2018. The soil samples were collected at the beginning of November 2018 (when the vegetation period was ended) near each of ceramic cups of the lysimeters. Until the tests were performed soil and water samples were stored in the dark, cold place (T = 4 °C). All determinations were performed within 1 week of the samples’ arrival in the laboratory.

### Determination of physicochemical parameters of soil and heavy metal concentrations in soil solutions

In all the soil samples, the grain size distribution was determined by Casagrande’s hydrometer method modified by Prószyński (PN-ISO, 11277: 2009). The following fractions were distinguished: sand (particle size 0.05–2.0 mm), silt (particle size 0.002–0.05 mm), and clay (particle size below 0.002 mm). After determining the share of granulometric fractions, individual soil types were determined in accordance with the United States Department of Agriculture (USDA) classification. Dry matter content in soil (soil moisture, SM) was established gravimetrically according to PN-ISO 11465: 1999. Soil pH were established by using glass electrode in a 1:5 (w/w) suspension of soil in water (pH in H_2_O) and in 1 mol L^−1^ potassium chloride solution (pH in KCl) (PN-ISO, 10390: 2005) (Table 1). Exchangeable cations (CEC) were determined using 1 M NH4Ac by AAS detection (Krzaklewski et al., 2004). The amount of organic carbon in soils was assessed by oxidation of organic matter with a mixture of potassium dichromate and sulfuric acid (PN-ISO 14235: 2003). Soil total N was determined by the Kjeldahl method (Bremner & Mulvaney, 1982).

**Table 1:** Physicochemical properties of soils (mean ± SD)

Soil layer	Site	Soil type^a^	Corg	N	SM	CEC	pH	
			(% dw)		(% dw)	(cmol_c_ kg^−1^)	H_2_O	KCl
A	OR	Silt loam	1.7 ± 0.2	0.1 ± 0.1	37.8 ± 8.7	8.7 ± 1.4	4.3 ± 1.1	4.5 ± 0.7
	SR	Silt loam	2.3 ± 0.5	0.2 ± 0.1	26.3 ± 4.7	11.4 ± 3.0	5.0 ± 1.2	4.0 ± 0.4
	CL	Silt loam	2.9 ± 0.5	0.2 ± 0.1	30.4 ± 7.6	11.9 ± 2.6	4.5 ± 1.1	4.3 ± 0.9
	KB	Silt loam	6.1 ± 1.2	0.1 ± 0.1	34.0 ± 3.4	9.1 ± 2.6	4.4 ± 1.2	4.1 ± 1.1
	GD	Silt loam	7.5 ± 1.4	0.4 ± 0.2	45.6 ± 7.3	15.8 ± 1.7	5.3 ± 1.4	4.3 ± 0.8
	MS	Silt loam	7.6 ± 1.7	0.2 ± 0.1	35.5 ± 3.6	9.3 ± 1.2	4.5 ± 0.9	4.0 ± 1.2
B	OR	Silt loam	0.7 ± 0.2	0.2 ± 0.1	22.3 ± 3.8	15.5 ± 3.9	4.5 ± 1.1	4.0 ± 1.1
	SR	Silt loam	0.4 ± 0.1	0.1 ± 0.1	20.4 ± 2.0	9.3 ± 1.8	5.0 ± 1.4	3.9 ± 1.2
	CL	Silt loam	1.4 ± 0.1	0.2 ± 0.1	22.6 ± 5.9	6.5 ± 0.7	4.9 ± 0.9	4.8 ± 1.1
	KB	Silty clay loam	2.2 ± 0.6	0.1 ± 0.1	28.0 ± 4.2	5.3 ± 0.8	5.2 ± 0.8	4.7 ± 0.7
	GD	Silt loam	1.4 ± 0.3	0.2 ± 0.1	28.5 ± 3.1	9.3 ± 1.0	5.2 ± 1.5	3.5 ± 0.5
	MS	Silt loam	3.1 ± 0.6	0.2 ± 0.1	31.1 ± 3.4	6.3 ± 0.8	6.7 ± 0.7	4.2 ± 0.9

^a^United States Department of Agriculture (USDA) classification

Abbreviations: *C*_*org*_ organic carbon, *SM* soil moisture

The total content of lead in the soil was determined, after ashing the soil in the furnace (450 °C) and digesting the ash with aqua regia, by inductively coupled plasma optical emission spectrometry (ICP-OES) technique using Thermo Scientific iCAP 6500 equipment. The quality assurance and quality control was performed by analyzing the standard samples of the known composition. Cation concentrations (Pb^2+^) in soil solutions have been determined directly in the samples by also using ICP-OES technique.

### Soil enzyme activity

Soil enzyme activity was determined in moist soil. The activity of acid and alkaline phosphatase (AcP and AlP), dehydrogenase (Deh), and urease (Ure) was measured in accordance with the methodology proposed by Schinner et al. (2012).

The activity of both phosphatases was examined by colorimetric method. This method is based on colorimetric estimation of p-nitrophenol (pNP) released when soil is incubated with buffered sodium p-nitrophenyl phosphate (pNPP) solution and toluene at 37 °C for 1 h. Activity of both enzymes was expressed in μmol pNP g^−1^ d in soil h^−1^. Absorbance was measured at a wavelength λ = 400 nm. MUB buffer with pH 6.5 for acid phosphatase (AcP) and pH 11 for alkaline (ALP) was used to optimize the reaction. CaCl_2_ (0.5 M) and NaOH (0.5 M) were used to brake reaction time.

Dehydrogenase activity (Deh) was determined by the method described by Casida et al. (1964). As a substrate, the triphenyltetrazolium chloride (TTC) was used. TTC was reduced to red-colored triphenylformazan (TPF). Dehydrogenase activity was expressed in μg TPF g^−1^ day in soil 16 h^−1^. Absorbance was measured at λ = 546 nm. Urease activity was determined colorimetrically based on determination of ammonium formation after the enzymatic urea hydrolysis at λ = 630 nm and expressed in μg N g^−1^ d in soil h^−1^.

### Soil microbial functional diversity — Biolog EcoPlates technique

Biolog EcoPlates are 96-well plates, containing three replicate sets of 31 different substrates, which are ecologically relevant and structurally diverse compounds. The selected substrates are widely used to assess functional diversity of soil microbial communities (Chojniak et al., 2015; Preston-Mafham et al., 2002). 

Ten grams of soil was shaken in 90 mL of distilled sterile water for 20 min at 25 °C. Then 150 μL of each sample was inoculated into each well of Biolog EcoPlates and incubated at 26 °C. The determinations have been performed by spectrophotometric measurements of absorbance used by sets of microorganisms of carbon substrates. The oxidation of carbon substrates was read using an automatic microplate reader, by measuring the intensity of the color change as a result of dye reduction—tetrazolium violet caused by microorganisms (Garland & Millis, 1991). Seventy-two hours was the shortest incubation time in which the highest variation between the examined objects was found. The average absorbance — AWCD (average well color development) — for each soil was calculated based on the absorbance for 31 substrates, less the absorbance for pure water, according to the formula: AWCD = Σ(n_i_)/31, where n_i_ is the absorbance of each substrate (Hu et al., 2011). The calculated AWCD value was used as a measure of the activity of soil microorganisms. The richness index (Rs) was calculated based on the number of carbon substrates that were used by the microorganisms. The microbial functional diversity index (H′) was calculated on the basis of Shannon–Wiener diversity index. In the calculations, a number of substrates and the utilization of an individual substrate by microorganisms were taken into account (Derry et al., 1999; Hu et al., 2011; Klimek et al., 2016).

### Measurements of soil respiration

After transporting to the laboratory, the soil was sieved (2 × 2 cm) in order to separate stones, invertebrates, and plant roots. Then, 200 g of soil was transferred to glass soil chambers and kept at 10 °C for 1 day. The chambers were open during the stabilization period. Soil respiration (CO_2_ efflux) was determined by using infrared gas analyzer LCProplus (ADC Bioscientific, UK) (measurement time, 1 h; air flow, 200 µmol min^−1^).

### Statistical analysis

The analysis of variance (ANOVA) was used in order to asses diversity in soil enzyme activity, soil respiration, and indexes of functional diversity of soil bacteria. Relationship between the selected parameters was assessed using the Pearson’s correlation coefficient. Outliers were discarded from the analysis. The following significance levels are indicated in the tables: *p < 0.05, **p < 0.01, ***p < 0.001. In order to illustrate the relationship between the physicochemical soil parameters and metabolic activity of soil microflora, the principal component analysis (PCA) was used. On the diagrams PC1 and PC2 components are shown, which present the largest variance. To perform the calculations, we used a Dell Statistica (data analysis software system), version 13.

## Results and discussion

### Lead concentrations in soil and soil solutions

Over the years, many authors have found increased values of heavy metals in the Beskidy mountain forests. In the research on soil humus collected in Czech part of the Beskidy Mountains bordering the Beskid Śląski and conducted in the early 2000s by Suchara and Sucharova (2002), it was found there was an increased deposition of trace elements like: Fe, Cu, Zn, Cd, As, and Pb. The authors emphasized the fact that this area was affected by local sources, mainly steel works as well as by air-borne pollutants transported long distances from domestic and foreign sources. In our study, the content of Zn, Cu, and Cd in the topsoil was similar to their content in the bedrock (Table 2). Already at the beginning of the twenty-first century, the emission of heavy metals in Poland was significantly reduced; thus, their deposition to ecosystems presumably decreased significantly which can explain the results obtained for these elements.

**Table 2:** The content of Zn, Cu, and Cd [mg kg^−1^ dw] in the soil (mean ± SD)

	Layer A			Layer B			Layer C (background)		
	Zn	Cu	Cd	Zn	Cu	Cd	Zn	Cu	Cd
OR	78.3 ± 10.6	17.5 ± 4.7	0.2 ± 0.1	96.8 ± 13.6	36.9 ± 8.8	0.2 ± 0.1	98.0 ± 5.4	51.1 ± 5.1	0.2 ± 0.1
SR	72.4 ± 3.5	20.2 ± 5.9	0.5 ± 0.1	78.9 ± 2.1	25.9 ± 2.7	0.5 ± 0.1	104.9 ± 10.8	37.6 ± 5.4	0.1 ± 0.1
CL	72.0 ± 10.1	17.7 ± 1.8	0.5 ± 0.1	63.2 ± 6.8	14.6 ± 2.3	0.5 ± 0.1	78.6 ± 9.6	23.3 ± 1.6	0.2 ± 0.1
KB	31.2 ± 6.4	8.4 ± 2.1	0.5 ± 0.2	25.0 ± 1.8	4.9 ± 0.7	0.4 ± 0.1	59.7 ± 9.3	28.2 ± 2.1	0.2 ± 0.1
GD	69.9 ± 11.9	20.6 ± 0.7	0.7 ± 0.1	87.7 ± 9.8	13.1 ± 4.9	0.3 ± 0.1	81.3 ± 3.4	22.8 ± 2.1	0.1 ± 0.1
MS	36.6 ± 13.8	10.9 ± 2.7	0.4 ± 0.1	32.5 ± 9.9	6.9 ± 0.9	0.3 ± 0.1	80.8 ± 9.3	15.4 ± 2.4	0.2 ± 0.1

On the other hand, pollutants such as heavy metals can persist for a long time in the environment. At the beginning of 2000s, high content of lead in the Beskid Śląski mountains was observed by Rrokicka-Kieliszewska et al. (2002) (238 and 361 mg Pb kg^−1^). However, it appears that the content of this element in soil is still elevated. In our research, its concentration in soil was from 23.5 to 180.0 mg Pb kg^−1^ dw (Fig. 3). For the MS site, the average lead content at all sites was close to 150.0 mgPb kg^−1^ dw defined as maximum lead level tolerable for soil biota (De Vries et al., 2007). Those elevated lead concentrations, combined with acidic soil and high content of organic matter, may have negative impact on soil microbiota. On the other hand, similar and higher concentrations of lead in the surface layers of soil were reported by other authors in other mountain ranges including the Karkonosze Mountains (Reimann et al., 2009; Szopka et al., 2013). In the case of the Karkonosze, as in the case of the Beskidy Mountains, the elevated lead content in the soil was probably influenced by industrial emissions. It is interesting that even now, despite the observed decreasing deposition of heavy metals into the environment, the biomonitoring studies performed on bryophytes still showed a greater deposition of trace elements in the Beskidy Mountains compared to the Karkonosze or other forests ecosystems in southern Poland (Kłos et al., 2015, 2018).

According to the standards introduced in September 2016 (Journal of Laws, 2016), the amount of lead in soil A layer did not exceed the admissible values (500 mg Pb kg^−1^ dw). However, according to the previously applicable Act (Journal of Laws, 2002) where permissible level for lead in soil was 100 mg Pb kg^−1^ dw, the lead concentration in the soils would be exceeded in GD and MS sites. 

**Fig. 2:**
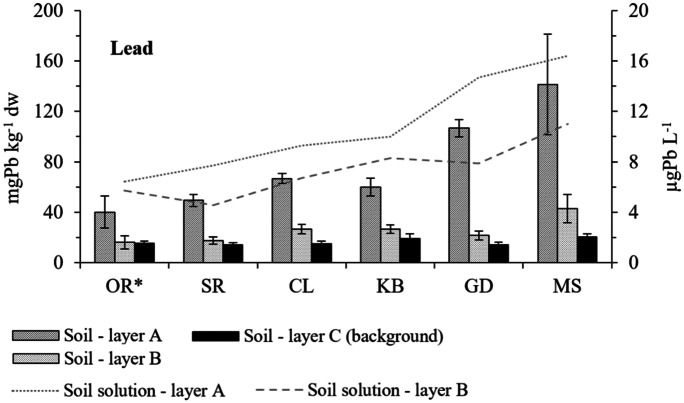
The content of lead in the soil and soil solution (mean ± SD). *Control site

More important than the amount of heavy metals in the soil is their content in dissolved form in soil solutions, because in this form these elements can move in the soil environment and have a negative impact on it. The content of the selected metals in soil solutions in topsoil (A layer) was in the range of 6.8–15.8 µg Pb L^−1^, and it was higher than that in B horizon (4.9–10.8 µg Pb L^−1^) (Fig. 2). Nearly 20 years ago on MS and SG sites, Staszewski et al. (2008) recorded lower amounts of lead and cadmium in soil solutions (2.9 µg Pb L^−1^ on SG and 10.9 µg Pb L^−1^ on MS sites). The conducted analysis showed that soil acidification and organic carbon content may have an impact on increasing the mobility of these elements in the soil environment (Table 3). The influence of pH on the mobility of metals was well documented by other authors (Kabala et al., 2014; Ramakrishnaiah & Somashekar, 2002).

**Table 3:** Pearson’s correlation coefficients between the concentration of lead cations in soil solution and selected soil parameters (*n* = 24; *p < 0.05, **p < 0.01, ***p < 0.001)

		Pb	pH_H2O_	Corg	SM	CEC
Pb^2+^	Layer A	0.77***	− 0.45*	0.75***	0.32	0.20
	Layer B	0.83***	− 0.58**	0.72***	0.37	− 0.32

### Soil enzyme activity

Soil enzyme activity can be taken as an indicator of heavy metal burden on the soil environment. In the area affected by heavy metal contamination like post-industrial sites, their activity is usually lower than that in clean areas (Borgulat, 2017; Gucwa-Przepióra et al., 2016; Nadgórska-Socha et al., 2006, 2013). In the present study, it was found that despite the variation in lead content in the topsoil at the research sites, there were no significant differences in Deh, Ure, and AlP activities (Table 4). However, it should be pointed out that the values obtained for lead concentration in the soil were at a lower level than those in heavily polluted areas (see the previous paragraph). In the case of the rest of analyzed heavy metals, their contents were close to the background values (Table 2). Therefore, it cannot be excluded that in the situation of moderate loading of the soil environment with heavy metals, e.g. lead, due to the adaptability of microorganisms, the activity of some soil enzymes did not change significantly. The other possible reason for the lack of differences in the mentioned soil enzyme activity is that the long-term presence of lead in the soil may have led to the diversification of microbial populations and the development of microorganisms resistant to its toxic effects (Kizylkaya et al. 2004, Ciarkowska et al., 2014).

**Table 4:** Average values of the analyzed parameters of microbial activity obtained for selected sites (mean ± SD)

	OR*	SR	CL	KB	GD	MS	Mean	SD		ANOVA	
										*F*	*p*-value
Layer A											
AlP (mg pNP g^−1^ dw h^−1^)	0.37 ± 0.26a	0.36 ± 0.08a	0.41 ± 0.18a	0.38 ± 0.22a	0.22 ± 0.11a	0.28 ± 0.04a	0.34	0.07		0.7	0.635
AcP (mg pNP g^−1^ dw h^−1^)	**0.76 ± 0.08a**	**0.85 ± 0.44a**	**1.04 ± 0.37b**	**0.59 ± 0.06a**	**0.57 ± 0.15a**	**0.54 ± 0.05a**	**0.73**	**0.20**		**5.9**	0.002**
Deh (μg TPF g^−1^ 16 h^−1^ dw)	0.45 ± 0.10a	0.74 ± 0.60a	0.69 ± 0.50a	1.05 ± 0.40a	0.99 ± 0.31a	0.66 ± 0.31a	0.76	0.22		1.6	0.209
Ure (μg N g^−1^ dw)	40.7 ± 12.3a	40.4 ± 3.2a	46.2 ± 9.5a	36.5 ± 0.9a	35.2 ± 5.0a	41.1 ± 4.6a	40.0	3.9		1.1	0.378
AWCD (OD)	**79.7 ± 17.3b**	**122.9 ± 5.9c**	**136.9 ± 35.6c**	**65.8 ± 15.5ab**	**44.7 ± 7.3ab**	**28.4 ± 8.2a**	**79.7**	**42.9**		**13.7**	< 0.001***
H″	**3.1 ± 0.1b**	**3.1 ± 0.1b**	**2.9 ± 0.3b**	**3.3 ± 0.1b**	**2.6 ± 0.1a**	**2.6 ± 0.1a**	**2.9**	**0.3**		**5.4**	**0.003**
Rs	**27.6 ± 1.4bc**	**23.8 ± 0.9abc**	**25.9 ± 5.5bc**	**29.2 ± 0.9c**	**19.9 ± 0.9ab**	**19.1 ± 1.8a**	**24.2**	**4.1**		**6.7**	0.001**
Sres (mg CO_2_-C kg^−1^soil h^−1^)	**3.6 ± 0.7a**	**4.5 ± 0.9a**	**4.6 ± 0.3a**	**5.0 ± 0.9a**	**8.3 ± 0.2b**	**3.7 ± 0.8a**	**5.0**	**1.7**		**18.2**	< 0.001***
Layer B											
AlP (mg pNP g^−1^ dw h^−1^)	0.11 ± 0.08a	0.05 ± 0.02a	0.1 ± 0.06a	0.08 ± 0.11a	0.29 ± 0.05a	0.15 ± 0.07a	0.13	0.09		1.7	0.179
AcP (mg pNP g^−1^ dw h^−1^)	0.49 ± 0.14a	0.29 ± 0.08a	0.49 ± 0.29a	0.36 ± 0.12a	0.39 ± 0.06a	0.34 ± 0.04a	0.39	0.08		1.5	0.236
Deh (μg TPF g^−1^ 16 h^−1^ dw)	**0.04 ± 0.10ab**	**0.33 ± 0.10a**	**0.38 ± 0.10abc**	**0.95 ± 0.10 cd**	**0.74 ± 0.20d**	**0.85 ± 0.10bc**	**0.55**	**0.35**		**3.1**	0.033*
Ure (μg N g^−1^ dw)	28.3 ± 9.1a	16.3 ± 6.4a	22.3 ± 1.5a	14.8 ± 9.2a	32.9 ± 6.3a	20.6 ± 6.3a	22.5	7.0		1.5	0.242
AWCD (OD)	**90.8 ± 0.7a**	**67.8 ± 8.9b**	**73.6 ± 15.6ab**	**74.6 ± 17.8b**	**27.0 ± 6.0b**	**28.7 ± 13.6ab**	**60.4**	**26.4**		**5.8**	0.002**
H'	**1.2 ± 0.1a**	**0.9 ± 0.1bc**	**1.2 ± 0.1bc**	**1.3 ± 0.1bc**	**0.9 ± 0.1c**	**0.9 ± 0.2ab**	**1.1**	**0.2**		**5.0**	0.005**
Rs	**22.2 ± 1.2ab**	**20.6 ± 0.3 cd**	**19.4 ± 0.2bcd**	**18.5 ± 3.7abc**	**11.7 ± 1.8d**	**17 ± 2.3a**	**18.2**	**3.7**		**11.6**	< 0.001***
Sres (mg CO_2_-C kg^−1^soil h^−1^)	**0.8 ± 0.1a**	**1.7 ± 0.2a**	**1 ± 0.5b**	**2.4 ± 0.8b**	**3.6 ± 0.8b**	**3.1 ± 0.8c**	**2.1**	**1.2**		**6.3**	0.002**

Abbreviations: *Deh* dehydrogenase, *Ure* urease, *AlP* alkaline phosphatase, *AcP* acid phosphatase, *H′* microbial functional diversity index, *Rs* richness index,

*AWCD* average well color development. *p values < 0.1; **p values < 0.05; ***p values < 0.001

Other results were obtained for acid phosphatase (AcP) activity in the topsoil, for which significant differences were found between the selected sites. Table 1 presents the physicochemical properties of soils. All analyzed samples of layer A of soil can be classified as silt loam in accordance with the USDA classification. It therefore appears that the granulometric composition of the soil should not be one of the factors that could influence the variability of soil microbial activity parameters. The results obtained for AcP can be explained by the variability of two main factors. It was found that AcP activity in topsoil was positively correlated with soil pH (Table 5). The optimum pH of the activity of acid phosphomonoesterases is within a range of 4.0 to 6.5 (Rejsek et al., 2012). Herbien and Neal (1990) found that the optimal pH for phosphatase activity in forest soil was 4.9. At the SG and MS sites, a very acidic soil was found (pH < 4.0), which could have had a negative effect on the AcP activity. The pH close to optimal was found at the CL site where the highest activity of this enzyme was found.

**Table 5:** Pearson correlation coefficient between selected parameters (*n* = 24; *p < 0.05, **p < 0.01, ***p < 0.001)

		A layer							
		AlP	AcP	Deh	Ure	AWCD	H'	Rs	Sres
pH_H2O_		0.29	0.44*	− 0.13	− 0.13	0.43*	0.50*	0.46*	− 0.43*
pH_KCl_		0.18	0.29	− 0.27	0.18	0.40	0.59**	0.54**	− 0.46*
Corg		− 0.18	− 0.56**	0.34	− 0.29	− 0.63**	− 0.54**	− 0.55**	0.51*
N		− 0.02	− 0.30	0.34	− 0.35	− 0.43*	− 0.61**	− 0.50*	0.74***
SM		− 0.23	− 0.26	0.20	− 0.05	− 0.23	− 0.35	− 0.32	0.44*
CEC		0.12	0.04	0.07	− 0.38	− 0.16	− 0.34	− 0.51**	0.55**
Pb		− 0.18	− 0.44*	0.10	− 0.18	− 0.60**	− 0.68***	− 0.71***	0.22
Pb^2+^		− 0.26	− 0.46*	0.06	− 0.23	− 0.66***	− 0.55**	− 0.62**	0.38
	B layer								
	AlP		AcP	Deh	Ure	AWCD	H'	Rs	Sres
pH_H2O_	− 0.13		0.19	0.47*	0.12	− 0.08	− 0.11	− 0.01	− 0.41*
pH_KCl_	− 0.32		0.01	0.15	0.04	0.14	0.30	0.16	− 0.37
Corg	0.36		− 0.15	− 0.30	− 0.03	− 0.03	− 0.26	− 0.34	0.53**
N	0.23		− 0.10	− 0.06	0.02	− 0.09	− 0.20	0.09	0.14
SM	0.10		− 0.38	0.01	− 0.08	− 0.16	− 0.25	− 0.21	0.74***
CEC	− 0.38		− 0.16	0.30	− 0.23	− 0.01	0.11	0.15	− 0.09
Pb	0.19		− 0.23	− 0.21	0.01	0.00	− 0.22	− 0.44*	0.34
Pb^2+^	0.19		− 0.15	− 0.19	− 0.25	0.15	0.10	− 0.17	0.49*

Abbreviations: *Deh* dehydrogenase, *Ure* urease, *AlP* alkaline phosphatase, *AcP* acid phosphatase, *H*′ microbial functional diversity index, *Rs* richness index, *AWCD* average well color development, *Corg* organic carbon, *Sres* soil respiration, *SM* soil moisture

On the other hand, the analysis carried out showed a negative relationship between AcP activity and the content of lead and Pb^2+^ ions in the soil solution. Lead ions may also have significantly contributed to the inhibition of AcP activity. The toxic effects of metal ions on enzyme structures are due to the masking of catalytically active groups by the ions, resulting in the denaturation of active enzyme proteins (Gianfreda et al., 2005). However, it should be emphasized that AcP activity on all examined fields was relatively high (A layer: 0.50–1.31 mg pNP g^−1^ dw h^−1^). For example in Borgulat’s research (2017) conducted in pine-spruce forest near the Miasteczko Śląskie zinc smelter, the AcP activity in topsoil was in the range 0.001–0.014 mg pNP g^−1^ dw h^−1^. On sites located in spruce forests subjected to emissions from Huta Katowice metallurgical plants, the activity of the AcP was 0.006–0.031 mg pNP g^−1^ dw h^−1^. In the last century, these two industrial plants were among the biggest emitters of air pollutants in Upper Silesia, and the heavy metal contamination of soil surface near these plants was at very high level.

In the subsoil (layer B) lower activity of soil enzymes was found (Table 4) but the potential effect of lead on their activity due to its lower content than in topsoil was presumably small. Among the selected soil enzymes, significant differences in DeH activity were found between the selected sites. The determination of dehydrogenase activity in soil is an indicator of the intensity of the respiratory metabolism of microorganisms such as soil bacteria and actinomycetes (Januszek et al., 2015). Among others Mocek-Płóciniak (2006) indicated that the dehydrogenases (DeH) are good biomarkers of soil contamination with lead. However, the analyses carried out showed that the activity of this enzyme in soil collected in the Beskidy mountain forests was probably more influenced by soil pH than by lead content. In layer B of the soil, a significant positive correlation was found between soil pH and DeH activity (Table 5). Interestingly, no such correlation was found for layer A of the soil. However, in layer A the average pH values ranged from 4.3 to 5.3 (SD = 0.39), while in layer B slightly higher pH values were obtained (4.5–6.7) and the scatter of obtained results was larger (SD = 0.76). Fernandez-Calvino et al. (2010) also found a significant positive correlation between soil pH and DeH activity, and noted that acidic pH (of about 4.9 in water) negatively affected the activity of this enzyme. In the studies by Włodarczyk et al. (2002) and Ros et al. (2003), the optimum values for DeH were pH 7.1 and pH 7.6–7.8, respectively. Therefore, it seems that the soil pH values in the soil B layer on some sites (MS and GD) were closer to the optimum for DeH than in A layer which resulted in a statistically significant correlation between those parameters.

### Functional diversity of soil bacteria and soil respiration

**Fig. 3:**
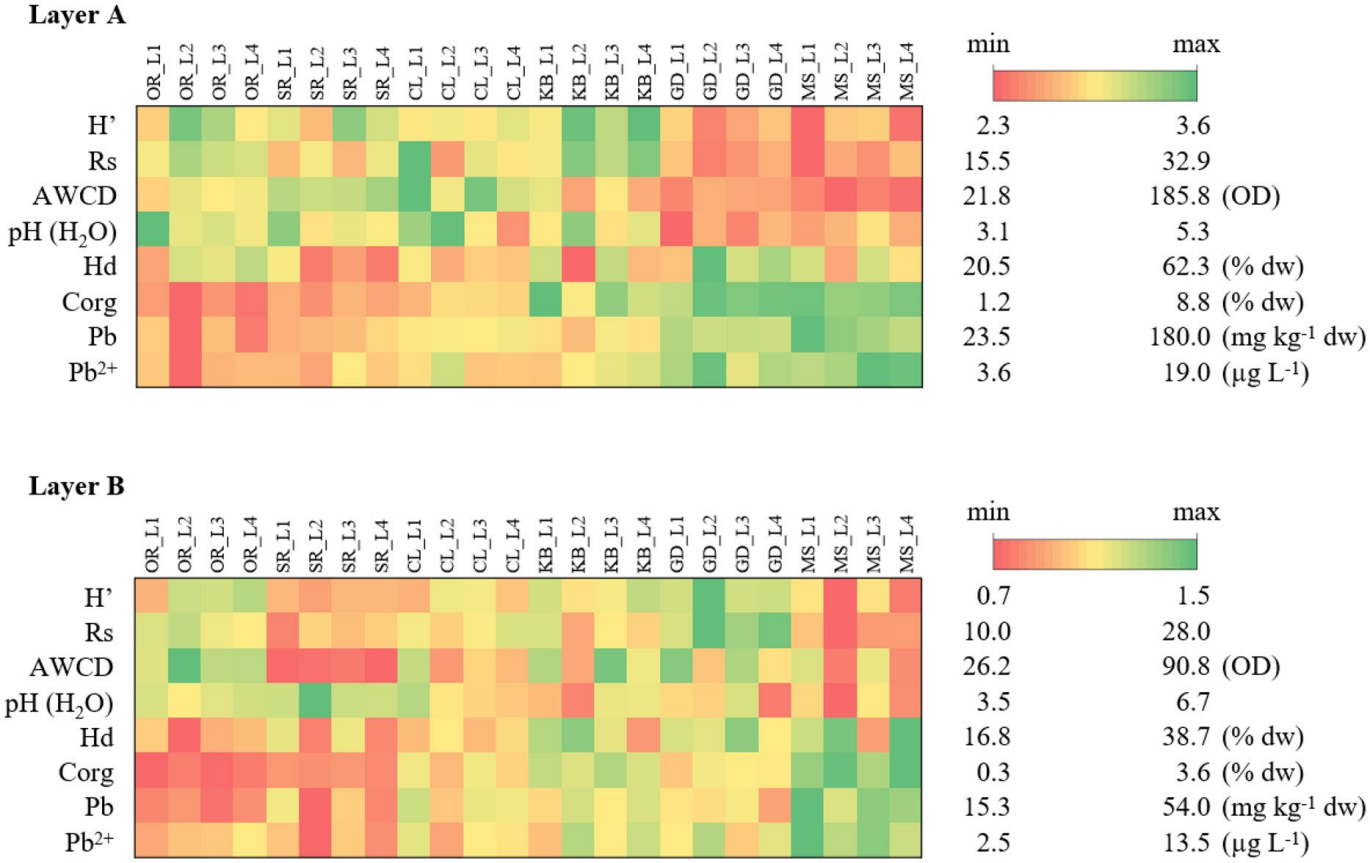
Selected parameters of metabolic activity of soil microorganisms determined using Biolog EcoPlate technique and soil pH, lead content in soil and in soil solutions (layer A)

The negative influence of heavy metals, including lead, on the functional diversity of soil microorganisms assessed by the Biolog EcoPlate technique was reported by many researchers (Kuźniar et al., 2018; Roane & Kellogg, 1996; Teng et al., 2008; Xie et al., 2016). This method was used due to the repeatability of the results and the availability of literature data. In this research it was noted that microbes inhabiting the topsoil environment on sites SG and MS, which had a relatively high lead content in topsoil (A layer), were characterized by statistically significant lower microbial functional diversity index (H′) and low metabolic activity (AWCD) (Table 4). The AWCD index provides information on the whole metabolic activity of microorganisms in the soil environment (Gomez et al., 2004) and reflects metabolic profiles of the soil microbial community, which could be affected by such pollutants as heavy metals (Fazekašová & Fazekaš, 2020; Teng et al., 2008). In this research, a large variation of this parameter was found (Fig. 3). Both AWCD and H′ correlated negatively with the presence of lead in soils and also with other soil characteristics like pH and content of organic carbon (Table 4). The acidic pH is considered to be one of the most important factors affecting the bioavailability of heavy metals (Zwolak et al., 2019) as confirmed by our research — in the case of low pH, a higher content of lead cations was found in the soil solutions (Fig. 3). Literature data indicates the influence of the presence of heavy metals on growth, morphology, and microbial metabolism, which leads to the decrease in the functional diversity of ecosystems (Hassan et al., 2013). The harmful effect of the presence of lead on the metabolism of microorganisms found in soil was demonstrated in the research of Niklińska et al. (2006). However, the authors stated that the low bioavailability of heavy metals may have influenced their results. In the present study, high mobility of lead was found so their potential impact on soil metabolism may indeed have occurred. In the subsoil (layer B), the lead content was at a lower level than in the topsoil (layer A) what could indicating its anthropogenic origin in the environment (Fig. 2). However, the presumed effect of lead on microbial activity, due to its relatively low content, was presumably negligible. In the case of AWCD, H′ and Rs parameters, their values were found to be lower in the layer B, which could be influenced by the lower amount of organic matter and lower soil moisture. The soil pH in layer A and B was at a similar level. Negative correlation (*r* =  − 0.44, *p* = 0.032) between the soil lead content and the Rs index indicating the potential use of different substrates by microorganisms was observed, but for the other parameters, no significant relationships were found.

**Fig. 4:**
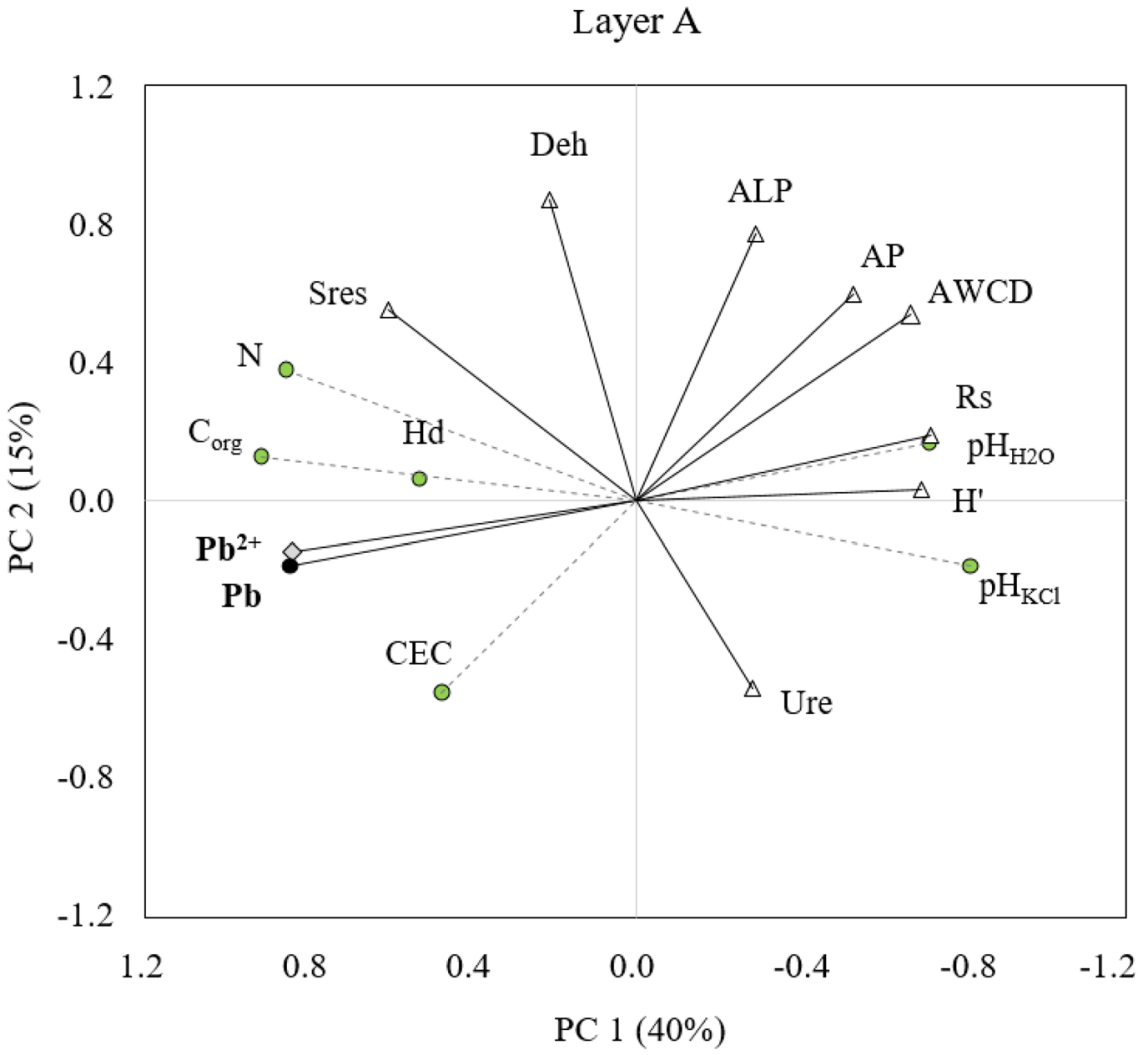
Principal component analysis (PCA) of soil physicochemical properties and indicators of soil microbiological activity (layer A)

**Fig. 5:**
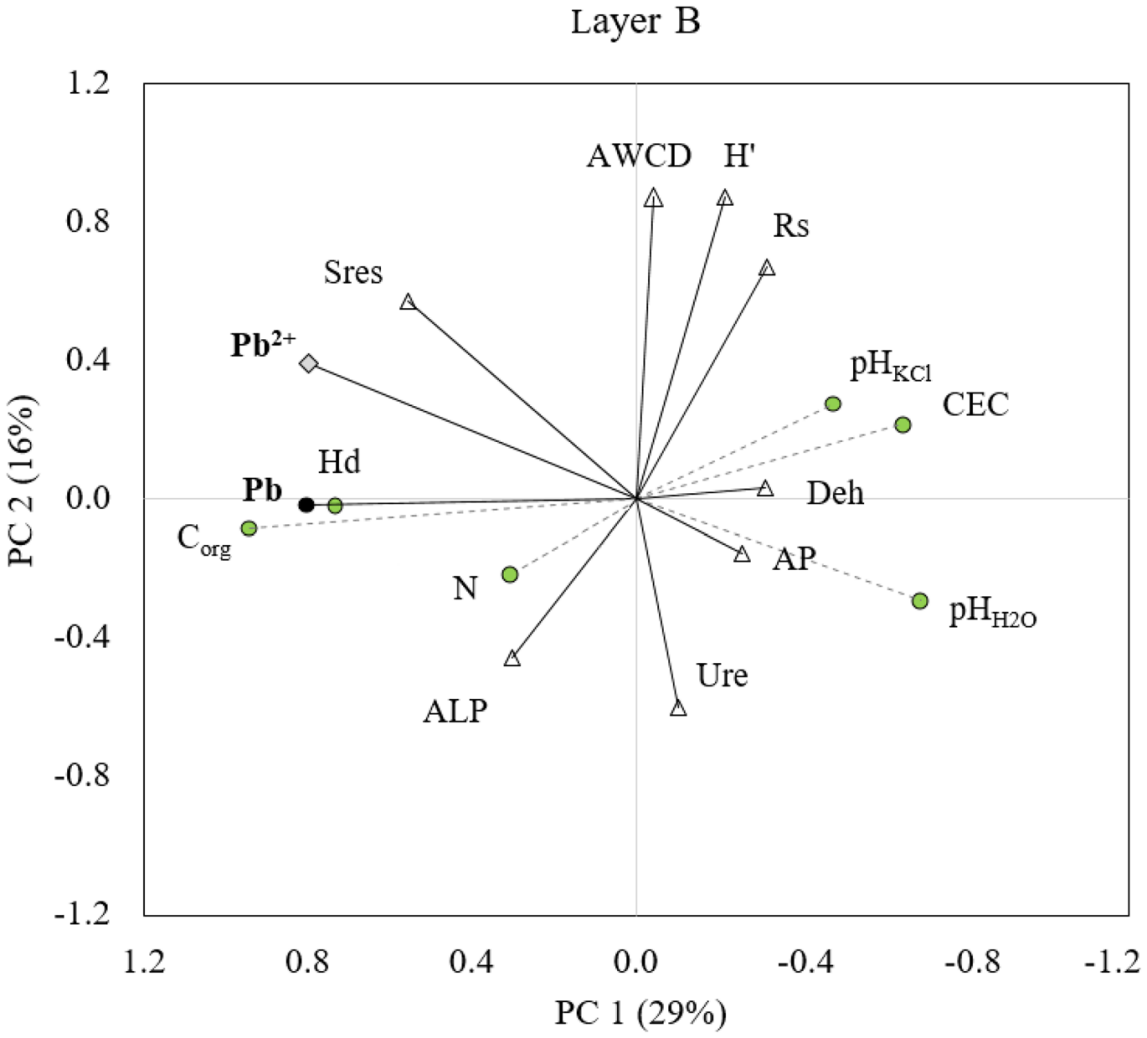
Principal component analysis (PCA) of soil physicochemical properties and indicators of soil microbiological activity (layer B)

Other researchers (Chodak et al., 2013) claimed that the use of soil respiration in the studies of the effects of heavy metals on forest soils might be difficult due to confounding influences of other environmental factors like pH and soil moisture. Our results confirm these observations. It should also be emphasized that the content of lead in the tested soil was moderately high — lower than those obtained in forests localized near urban areas and around industrial plants (Borgulat, 2017; Pająk, 2016; Rusinowski et al., 2019), which additionally made it difficult to identify significant differences. In our research, no significant relationships were found between the lead content in topsoil and the rate of soil respiration. The organic carbon (Corg) is a major source of energy for soil microorganisms, so the positive relationship between soil respiration (Sres) and its content should not be surprising (Table 5). PCA (Figs. 4 and 5) and the correlation analysis showed that the pH, carbon source, soil moisture, and amount of nitrogen were the most essential factors which had influence on soil respiration. Higher intensity of soil respiration was found for A layer (3.3–7.9 mg CO_2_-C kg^−1^soil h^−1^) than for layer B (0.5–4.0 mg CO_2_-C kg^−1^soil h^−1^) which was also influenced by the higher content of organic matter in the topsoil.

## Conclusions

The study found that elevated lead content in the soil environment, especially at low soil pH, may affect soil metabolism in forest ecosystems of the Beskidy mountain forests. In the case of the other heavy metals (Zn, Cu, and Cd), their content in the topsoil was at levels similar to those in the parent rock and their presence should not affect the metabolic activity of microflora. It seems that Biolog EcoPlates technique is the more sensitive method than soil enzyme analysis and it is more adequate for the assessment of soil environment pollution with heavy metals. The above results of the research carried out on the basis of various research methods indicate the need for a comprehensive study of the soil environment and the changes occurring in it under the influence of anthropopression.

## Availability of data and materials 

The dataset used and/or analyzed during the current study are available from the corresponding author on reasonable request. All bibliographical references analyzed during this study are included in this published article.
